# Probable depression and its correlates among undergraduate students in Johannesburg, South Africa

**DOI:** 10.3389/fpsyt.2023.1018197

**Published:** 2023-02-16

**Authors:** Jeremy Croock, Mafuno G. Mpinganjira, Kaashifa Gathoo, Robyn Bulmer, Shannon Lautenberg, Qhayiyakazi Dlamini, Pfanani Londani, Azola Solontsi, Chanel Stevens, Joel M. Francis

**Affiliations:** ^1^The Unit for Undergraduate Medical Education, School of Clinical Medicine, Faculty of Health Sciences, University of the Witwatersrand, Johannesburg, South Africa; ^2^Department of Family Medicine and Primary Care, School of Clinical Medicine, Faculty of Health Sciences, University of the Witwatersrand, Johannesburg, South Africa

**Keywords:** depression – epidemiology, university students and an educational program, screening, Patient Health Questionnaire (PHQ-2), correlates and predictors

## Abstract

**Background:**

Depression is associated with high rates of morbidity and mortality. Globally, depression is higher among university students than the general population—making it a significant public health problem. Despite this, there is limited data on the prevalence in university students in the Gauteng province, South Africa. This study determined the prevalence of screening positive for probable depression and its correlates among undergraduate students at the university of the Witwatersrand, Johannesburg, South Africa.

**Methods:**

A cross-sectional study, using an online survey was conducted among undergraduate students at the University of the Witwatersrand in 2021. Patient Health Questionnaire (PHQ-2) was used to assess the prevalence of probable depression. Descriptive statistics was computed and conducted bivariate and multivariable logistic regression to identify factors associated with probable depression. Age, marital status, substance use (alcohol use, cannabis use, tobacco use, and other substance use) were included in the multivariable model apriori determined confounders and other factors were only added if they had a *p*-value <0.20 in the bivariate analysis. A *p*-value of 0.05 was considered statistically significant.

**Results:**

The response rate was 8.4% (1046/12404). The prevalence of screening positive for probable depression was 48% (439/910). Race, substance use, and socio-economic status were associated with odds of screening positive for probable depression. Specifically reporting white race (adjusted OR (aOR) = 0.64, 95% CI: 0.42, 0.96), no cannabis use (aOR = 0.71, 95% CI: 0.44–0.99), higher spending power in the form of having the most important things but few luxury goods (aOR = 0.50, 95% CI: 0.31, 0.80) and having enough money for luxury goods and extra things (aOR = 0.44, 95% CI: 0.26–0.76) were associated with lower odds of screening positive for probable depression.

**Discussion:**

In this study, screening positive for probable depression was common among undergraduate students at the University of the Witwatersrand, Johannesburg, South Africa and associated with sociodemographic and selected behavioral factors. These findings call for strengthening the awareness and use of counselling services among undergraduate students.

## Introduction

Depression is a major mental health illness that goes largely undiagnosed. About 10% of the world’s population will experience depression in their lifetime, making it one of the most common mental health illnesses (1, 2). About 30 million people in Africa suffer from depression (3) and in South Africa (SA), 1 in 3 people suffer from either depression, anxiety or a substance use disorder. Despite this, only about 22.6% of those diagnosed receive treatment, mostly due to resource deprivation, stigma, and cultural beliefs around mental health illnesses (4–6).

Globally, the reported prevalence of depression amongst university students is high, with up to 21% of students suffering from major depressive disorder (MDD) (7, 8). In SA, it is estimated that 24.2% of university students suffer from mild depression, and 12.4% suffer from moderate to severe depression (9)—which is higher than the global prevalence, and thus deserving attention and more efforts (2, 9).

Mental health is particularly vulnerable to traumatic events, and the economic and social consequences that come with them. The traumatic events range from natural disasters, epidemics and political unrest (10). The recent coronavirus-2019 (COVID-19) pandemic represents one of those traumatic events, which together with the policies created to counteract the spread, has increased the burden of depressive symptoms, with augmented stratification amongst lower income and poor-resource exposed groups of individuals (11).

Students with depression have worse academic outcomes and low productivity (12), which further exacerbates depressive symptoms. These students are also more likely to struggle with alcohol abuse in their adulthood (13). Depression, even subclinical, has also been associated with high rates of suicide (14). Thus, it is of vital importance that students with depression are screened and diagnosed early and appropriately, to allow for adequate treatment.

Identifying the prevalence of depressive symptoms, as well as factors that are associated with depression, could assist in dealing with the illness, identifying at-risk groups earlier, and allow for measures to be put in place to target these specific factors. Students are susceptible to depression due to various factors in both their daily and academic lives. These factors include financial stressors, academic performance and exam stress, and changes in social roles and relationships (7, 15, 16). Moreover, and in relation to the issues of stigma and discrimination mentioned earlier, these factors contribute to students not seeking help, for fear of being judged or discriminated against (6). Previous study among undergraduate students in South Africa reported highest levels of depression, stress, and anxiety and these were associated with financial status and social life (17). It is therefore critical to ensure students have access to mental health services for screening and early treatment. Our study assessed the prevalence of screening positive for probable depression using PHQ-2 and its correlates among undergraduate students at the University of the Witwatersrand in Johannesburg, South Africa to inform potential interventions such as strengthening the awareness and use of counselling services for undergraduate students.

## Materials and methods

### Study design

A cross-sectional study, using an online survey was conducted among registered undergraduate students at the University of the Witwatersrand in February–April 2021.

### Study setting and population

Currently there are 28 universities in South Africa, of which 5 are in the Gauteng province. There are two in Johannesburg (Gauteng)—the University of Johannesburg and the University of the Witwatersrand. Wits University offers undergraduate courses in 5 faculties. In February–April 2021, University of the Witwatersrand had 12404 registered undergraduate students. All registered undergraduate students were eligible to participate in the study.

### Sample size and sampling

The estimated minimum sample size required for sufficient power was 820 students—assuming the prevalence of reported depressive symptoms of 24% (Bantjies et al. (9), a marginal error of 4% (Hajian-Tilaki (18), and a response rate of 50% and the undergraduate student population of 20,000. All undergraduate students registered at the time of the survey were invited to participate in the study.

### Data collection procedures

Data was collected using the Patient Health Questionnaire (PHQ-2) (19). The questions were embedded into REDCap (20) for an online survey. The University of the Witwatersrand registrar’s office shared the invitation email with study information and link to the online survey to all registered (*n* = 12404) undergraduate students. This survey was distributed first to Faculty of Health Sciences undergraduate students on 11 February 2021 and then to all other faculties undergraduate students on 26 April 2021. The distribution of the survey dates was advised by the University registrar.

Participation in the study was voluntary and participants had to provide informed consent by responding to the consent questions prior to accessing all survey questions. Data collection was anonymous, we did not collect any identifying information. Participants were allowed to complete the survey at their convenience and all responses were kept confidential only study team including the supervisors had access to the data.

### Ethical considerations

This study was carried out according to ethical principles for medical research involving human subjects (Declaration of Helsinki). Ethical approval was obtained from the University of the Witwatersrand Human Research Ethics Committee (HREC), ethical approval number MED19-08-0178, and approval to conduct the study was obtained from the university and relevant faculties. Prior to the commencement of the study, the questionnaire was piloted by having the study team complete it. Due to the sensitive nature of this study on depression, a mental health illness, privacy of participants was maintained, together with the confidentiality of their responses. The contact details of the South African Depression and Anxiety Group (SADAG), the South African Suicide Hotline (SACH) and the Wits University Counselling Careers and Development Unit (CCDU) were provided, to encourage participants to contact them should they suspect they are depressed.

### Study variables

#### Outcome

The outcome of interest was screening positive for probable depression on PHQ-2. The PHQ-2 scores range from 0 to 6 and a score of ≥3 is screening positive for probable depression (19). The PHQ-2 consistency in this study population was 77.3%.

#### Exposures

Exposures were sociodemographic factors such (age, race, area of residence, type and conditions of dwelling, educational level, year of study, religious beliefs, and marital status) and other factors hypothesized to contribute to depression such as financial stress, low socio-economic status, and substance abuse (alcohol use, tobacco use, other drug use). Erroneously, sex variable was omitted from our questionnaire sent out to the respondents.

#### Data management and analysis

The data management and analysis was conducted using Stata version 14. All variables were categorical and therefore computed proportions and report frequency and proportions for descriptive statistics. We used *Chi*^2^ test to determine the factors associated with screening positive for probable depression at bivariate analysis. We fitted a multivariable logistic regression to establish factors associated with screening positive for probable depression. Age, marital status, substance use (alcohol use, cannabis use, tobacco use, and other substance use) were included in the multivariable model apriori determined confounders and other factors were only added if they had a *p*-value <0.20 in the bivariate analysis (Supplementary Table 1). We report adjusted odds ratios (aOR), corresponding 95% Confidence Interval (CI) and *p*-values from the multivariable logistic regression output. A *p*-value < 0.05 was considered statistically significant.

## Results

**FIGURE 1:**
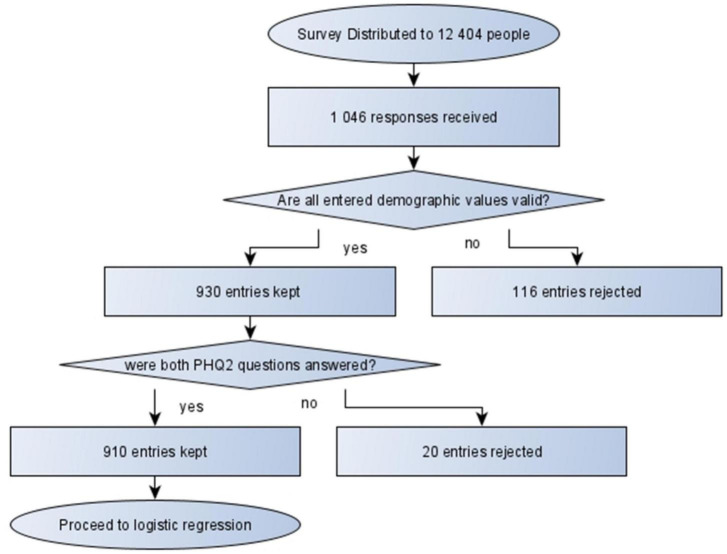
Flow of data collection through the study.

The survey link was distributed to 12,404 undergraduate students. 1,046 students responded to the survey equivalent to a response rate of 8.4%—such low response rates are common in online surveys (14). All entries (*n* = 116) with invalid demographic data or incomplete PHQ-2 were removed from the final analysis (Figure 1).

### Population characteristics

Most participants (*n* = 686, 73.8%) were 20 years, while the rest (*n* = 244, 26.2%) were between 18 and 19 years. Majority of the participants were Black African (*n* = 479, 52.4%), followed by White (*n* = 231, 25.3%), and with a minority reporting an unspecified race (*n* = 13, 1.4%). The highest proportion of participants reported their residence as suburban areas (*n* = 385, 42.3%), followed by urban areas (*n* = 359, 39.4%), with the least living in informal settlements (*n* = 18, 2.0%). Of these participants, majority lived in brick houses or flats (*n* = 843, 92.5%), and the rest in unspecified dwellings, shacks, wendy houses or backyard dwellings. Approximately 4 of 10 participants specified that they were able to afford the most important things but few luxury goods (*n* = 372, 41.6%), followed by those who were able to afford necessities and luxury goods (*n* = 321, 35.9%), with few having insufficient money for food (*n* = 15, 1.7%). Half of the participants were in their 3rd or 4th year of studies (*n* = 226, 24.8% and *n* = 223, 24.5% respectively). Most of the participants were single (*n* = 630, 69.2%). Reported alcohol use was common with 3 out 10 participants reporting alcohol use in the past week (*n* = 275, 30.2%). More participants reported cannabis use (*n* = 167, 18.45%) than tobacco use (*n* = 100, 11%) (Table 1).

**TABLE 1:** General characteristics of the University of Witwatersrand undergraduate students (*N* = 930) who participated in the survey February–May 2021.

Characteristic	Categories^a^	*N*	%
Age	18–19 years	244	26.2
	20 years and above	686	73.8
	Total	930	100.0
Race	Black African	479	52.4
	Colored	30	3.3
	Indian/Asian	161	17.6
	White	231	25.3
	Other	13	1.4
	Total	914	100.0
Residential area	Informal settlements	18	2.0
	Rural	116	12.7
	Urban	359	39.4
	Suburban	385	42.3
	other	33	3.6
	Total	911	100.0
Type of home	Shack	22	2.4
	Wendy house or backyard dwelling	17	1.9
	Brick house or flat	843	92.5
	Other	29	3.2
	Total	911	100.0
Income	We don’t have enough money for food	15	1.7
	We have enough money for food but not for other basic items such as clothes	73	8.2
	We have enough money for food and clothes, but we are short of many other things	114	12.7
	We have the most important things, but few luxury goods	372	41.6
	We have money for luxury goods and extra things	321	35.9
	Total	895	100.0
Highest qualification	High school diploma or equivalent	643	71.5
	Bachelor’s degree	188	20.9
	Honors degree	68	7.6
	Total	899	100.0
Year of study	1	181	19.8
	2	162	17.8
	3	226	24.8
	4	223	24.5
	5	64	7.0
	6	56	6.1
	Total	912	100.0
Religion	Christianity	590	64.9
	Judaism	23	2.5
	Islam	98	10.8
	Hinduism	31	3.4
	Buddhism	1	0.1
	Atheism	60	6.6
	Other	106	11.7
	Total	909	100.0
Marital status	Single	630	69.2
	Single in relationship	262	28.8
	Married	18	2.0
	Total	910	100.0
Alcohol use	Never	255	28.0
	In the past week	275	30.2
	In the past month	162	17.8
	In the past 6 months	138	15.1
	In the past 12 months	20	2.2
	Longer than 12 months ago	62	6.8
	Total	912	100.0
Tobacco use	Yes	100	11.0
	No	809	89.0
	Total	909	100.0
Cannabis use	Yes	167	18.4
	No	742	81.6
	Total	909	100.0
Other substance use	Yes	39	4.3
	No	867	95.7
	Total	906	100.0

^a^Varying total denominator due to missing values.

### Prevalence of probable depression and depressive symptoms

Almost half 437 (48.2%) of the participants were classified as having probable depression by PHQ-2. Participants mostly reported feeling “little interested or pleasure in doing things”—PHQ-1 on “several days” (*n* = 374, 41.1%). Similar findings were also obtained for PHQ-2 where participants reported “several days” when asked how frequently they felt down, depressed, or hopeless in the last two weeks (*n* = 411, 45.1%) (Table 2).

**TABLE 2:** Prevalence of probable depression and depressive symptoms using PHQ-2 among the University of Witwatersrand undergraduate students (*N* = 908) who participated in the survey February–May 2021.

PHQ-2 questions	Categories	*N*	%
Overall PHQ-2 status	No probable depression	471	51.8
	Probable Depression	439	48.2
Frequency of feeling little interested or pleasure in doing things	Not at all	162	17.8
	Several days	374	41.1
	More than half the days	183	20.1
	Nearly everyday	192	21.1
Frequency of feeling down, depressed, or hopeless over the past two weeks)	Not at all	166	18.2
	Several days	411	45.1
	More than half the days	183	20.1
	Nearly everyday	151	16.6

### Factors associated with probable depression

Factors associated with screening positive for probable depression were cannabis use and socioeconomic status. Specifically, participants reporting not using cannabis (OR = 0.67, 95% CI: 0.45–0.99), higher spending power both in the form of having the most important things but few luxury goods (OR = 0.51, 95% CI: 0.32, 0.81), and having enough money for luxury goods and extra things (OR = 0.45, 95% CI: 0.26–0.76) (Table 3) were associated with decreased odds of screening positive for probable depression.

**TABLE 3:** Factors associated with screening positive for probable depression (in multivariable analysis) among the University of Witwatersrand undergraduate students (*N* = 908) who participated in the survey February–May 2021.

Characteristic	Categories	*N*	%^a^	aOR^b^	95% CI	*P*-value^c^
Age	18–19 years	124	53.7	1.00		0.858
	20 years and above	313	46.6	0.94	0.59–1.49	
Race	Black African	259	54.5	1.00		0.083
	Indian/Asian	71	44.1	0.91	0.58–1.42	
	White	83	35.9	0.64	0.42–0.96	
	Other	26	60.5	1.35	0.69–2.65	
Income	We have enough money for food and clothes, but we are short of many other things	11	73.3	1.00		**0.018**
	We don’t have enough money for food	40	54.8	2.57	0.54–12.87	
	We have enough money for food but not for other basic items such as clothes	74	65.5	0.65	0.35–1.21	
	We have the most important things, but few luxury goods	178	48	0.50	0.31–0.80	
	We have money for luxury goods and extra things	125	38.9	0.44	0.26–0.76	
Year of study	1	94	52.2	1.00		0.062
	2	92	56.8	1.47	0.91–2.38	
	3	107	47.3	1.07	0.62–1.85	
	4	108	48.4	1.07	0.61–1.87	
	5	24	37.5	0.85	0.41–1.77	
	6	14	25.5	0.46	0.20–1.04	
Marital Status	Single	630	69.2	1.00		0.554
	Unmarried	262	28.8	1.21	0.87–1.69	
	Married	18	2	1.22	0.43–3.49	
Alcohol use	Never drunk alcohol	129	50.8	1.00		0.853
	In the past week	134	48.7	1.01	0.67–1.52	
	In the past month	75	46.3	0.95	0.60–1.49	
	In the past 6 months	101	46.1	0.86	0.58–1.28	
Tobacco use	Yes	60	60	1.00		**0.045**
	No	377	46.7	0.7	0.44–1.14	
Other substance use	Yes	39	4.3	1.00		0.533
	No	867	95.7	1.27	0.60–2.68	
Cannabis use	Yes	100	59.9	1.00		**0.045**
	No	338	45.7	0.71	0.44–0.99	

^a^Row percentage. ^b^Odds ratio adjusted for race, income level, year of study, marital status, tobacco use, other substance use, and cannabis use. ^c^Overall *p*-value for each factor (variable). Bold values represent the *p*-values < 0.05.

## Discussion

Our study sought to determine the prevalence of depressive symptoms and associated factors among undergraduate students at the university of the Witwatersrand, Johannesburg, South Africa. Overall, almost half of the study participants screened positive for probable depression. Probable depression was associated with sociodemographic and modifiable behavioral factors.

The prevalence of probable depression among undergraduate students in this study was high, but within a range of the reported prevalence of depression in individual studies included in a systematic review on depression among undergraduate university students in low- and middle-income countries, whereby the prevalence of depression ranged between 2.9 and 71% (21).

In previous studies, there has been mixed findings on the association of depression and sociodemographic and economic factors, for example, Akhatar et al., reported no association of depressive symptoms and socio economic status, however, Ibrahim et al. reported less depressive symptoms among students in high income families. In our study race and spending power were associated with less probable depression similar to findings from a study by Yakasai et al., among undergraduate students in South Africa (7, 17, 21, 22).

Our study shows that cannabis use was associated with increased odds of screening positive for probable depression, however, the literature on cannabis use and depression has had inconsistent findings, for example, in one study, cannabis use in adolescence correlated with depression in young adulthood (23) even though in this study it was not possible to determine causality by Moore et al., in their paper on the study conducted among prisoners cannabis use was associated with increased risk of any mental illnesses the association with depression alone was inconsistent (22, 24).

Substance use (alcohol and tobacco) are often used as coping mechanisms for individuals suffering from depression. In this study, reported alcohol use was common among the study participants but was not associated with screening positive for probable depression this is contrary to findings from other studies where excessive alcohol use was with depression (25). Even though we did not find association between tobacco use and screening positive for probable depression in this study other investigators have reported strong association between smoking and depression in adolescents and adults (26, 27).

Our findings should be interpreted considering a few limitations. First, we could not assess, adjust for and present probable depression by sex because this variable was erroneously omitted in the online link shared by the study participants, however, findings from other studies showed that the weighted mean average of depression prevalence was higher in female than male university students (29.6%, 95% CI: 29.2–30.1 vs 24.9%, 95% CI: 24.4–25.4) (7) and depressive disorders also followed a similar pattern of higher prevalence in females than males (28). In addition, previous studies, showed an association between gender and self-esteem and thus self-esteem was associated with depression (29). We could not assess these two aspects in this study. Furthermore, there other factors associated with depression among university students such as social connectedness with depression (30). Social connectedness and social cultural factors are critical issues, but they were beyond the scope of our study and therefore adds to the limitation of our work.

Second, due to social desirability, the study participants may have underreported behavioral factors, for example, substance use that could have biased our results toward the null. This issue was partly addressed by the survey being anonymous and online.

Third, the online survey is prone to selection bias and therefore only participants with access to internet could have responded to the survey, however, all students would have access to email and internet at the campus.

Fourth, this study was carried out during COVID-19 pandemic and the restrictions and therefore could have increased the reporting of depressive symptoms.

Fifth, the study findings could only be generalized for undergraduate students in Johannesburg, South Africa.

Lastly, because of the cross-sectional nature of the study it is not possible to determine the causal factors for probable depression.

In this study, screening positive for probable depression was common among undergraduate students at the University of the Witwatersrand, Johannesburg, South Africa and associated with sociodemographic and selected behavioral factors. Future studies should explore the role of information and social processing as mechanisms for the underlying psychological drivers of depression among university students (31, 32). In addition, these findings call for strengthening the awareness and use of counselling services among undergraduate students.

## Data availability statement

The raw data supporting the conclusions of this article will be made available by the authors, without undue reservation.

## Ethics statement

The studies involving human participants were reviewed and approved by University of the Witwatersrand (WITS) Human Research Ethics Committee (HREC), ethical approval number MED19-08-0178. Written informed consent for participation was not required for this study in accordance with the national legislation and the institutional requirements.

## Author contributions

JC, KG, RB, SL, QD, PL, AS, CS, and JF conceived and designed the study. JC, KG, RB, QD, PL, AS, CS, and JF participated in the data collection process. JC, MM, KG, RB, and JF analyzed the data. JC, MM, KG, RB, SL, QD, PL, AS, CS, and JF interpreted the findings. JC, MM, KG, and RB with the guidance of JF wrote the first draft of the manuscript. JC, MM, KG, RB, SL, QD, PL, AS, CS, and JF critically reviewed and approved the final draft of the manuscript. All authors contributed to the article and approved the submitted version.
