# Supplementary material for: Probable depression and its correlates among undergraduate students in Johannesburg, South Africa
Source: Front Psychiatry. 2023 Feb 16;14:1018197. doi: 10.3389/fpsyt.2023.1018197 (PMC9978096; doi:10.3389/fpsyt.2023.1018197)
Supplement: Supplementary file 1 [file Table_1.docx]

**Supplementary Table 1: Factors associated with screening positive for probable depression *(in bivariate analysis*) among the University of Witwatersrand undergraduate students (N=908) who participated in the survey February -May 2021**

| **Characteristic** | **Categories** | **PHQ 2 Screening status** | | | | |
| --- | --- | --- | --- | --- | --- | --- |
|  |  | No probable Depression | | Probable Depression | | P value^a^ |
|  |  | N | %^b^ | N | %^b^ |  |
| Age | 18-19 years | 107 | 46.3 | 124 | 53.7 | 0.062 |
|  | 20 years and above | 359 | 53.4 | 313 | 46.6 |  |
|  | Total | 466 | 51.6 | 437 | 48.4 |  |
|  |  |  |  |  |  |  |
| Race | Black African | 216 | 45.5 | 259 | 54.5 | **<0.001** |
|  | Indian/ Asian | 90 | 55.9 | 71 | 44.1 |  |
|  | White | 148 | 64.1 | 83 | 35.9 |  |
|  | Other | 17 | 39.5 | 26 | 60.5 |  |
|  | Total | 471 | 51.8 | 439 | 48.2 |  |
|  |  |  |  |  |  |  |
| Residence | Rural | 49 | 42.6 | 66 | 57.4 | 0.220 |
|  | Urban | 190 | 53.1 | 168 | 46.9 |  |
|  | Suburban | 202 | 52.6 | 182 | 47.4 |  |
|  | Other | 28 | 54.9 | 23 | 45.1 |  |
|  | Total | 469 | 51.7 | 439 | 48.3 |  |
|  |  |  |  |  |  |  |
| Income | We don’t have enough money for food | 4 | 26.7 | 11 | 73.3 | **<0.001** |
|  | We have enough money for food but not for other basic items such as clothes | 33 | 45.2 | 40 | 54.8 |  |
|  | We have enough money for food and clothes, but we are short of many other things | 39 | 34.5 | 74 | 65.5 |  |
|  | We have the most important things, but few luxury goods | 193 | 52.0 | 178 | 48 |  |
|  | We have money for luxury goods and extra things | 196 | 61.1 | 125 | 38.9 |  |
|  | Total | 465 | 52.1 | 428 | 47.9 |  |
|  |  |  |  |  |  |  |
| Qualifications | High school diploma or equivalent | 325 | 50.6 | 317 | 49.4 | 0.204 |
|  | Bachelor’s degree | 94 | 50.3 | 93 | 49.7 |  |
|  | Honors degree | 42 | 61.8 | 26 | 38.2 |  |
|  | Total | 461 | 51.4 | 436 | 48.6 |  |
|  |  |  |  |  |  |  |
| Year of study | 1 | 86 | 47.8 | 94 | 52.2 | **0.001** |
|  | 2 | 70 | 43.2 | 92 | 56.8 |  |
|  | 3 | 119 | 52.7 | 107 | 47.3 |  |
|  | 4 | 115 | 51.6 | 108 | 48.4 |  |
|  | 5 | 40 | 62.5 | 24 | 37.5 |  |
|  | 6 | 41 | 74.5 | 14 | 25.5 |  |
|  | Total | 471 | 51.8 | 439 | 48.2 |  |
|  |  |  |  |  |  |  |
| Religion | Christianity | 301 | 51.1 | 288 | 48.9 | 0.226 |
|  | Islam | 46 | 46.9 | 52 | 53.1 |  |
|  | Atheism | 38 | 63.3 | 22 | 36.7 |  |
|  | Other | 85 | 53.1 | 75 | 46.9 |  |
|  | Total | 470 | 51.8 | 437 | 48.2 |  |
|  |  |  |  |  |  |  |
| Marital status | Single | 322 | 51.2 | 307 | 48.8 | 0.658 |
|  | Single in a relationship | 138 | 52.9 | 123 | 47.1 |  |
|  | Married | 11 | 61.1 | 7 | 38.9 |  |
|  | Total | 471 | 51.9 | 437 | 48.1 |  |
|  |  |  |  |  |  |  |
| Alcohol use | I have never drunk alcohol | 125 | 49.2 | 129 | 50.8 | 0.723 |
|  | In the past week | 141 | 51.3 | 134 | 48.7 |  |
|  | In the past month | 87 | 53.7 | 75 | 46.3 |  |
|  | 6 months and more | 118 | 53.9 | 101 | 46.1 |  |
|  | Total | 471 | 51.8 | 439 | 48.2 |  |
|  |  |  | 0.0 |  | 0 |  |
| Tobacco use | Yes | 40 | 40.0 | 60 | 60 | **0.012** |
|  | No | 430 | 53.3 | 377 | 46.7 |  |
|  | Total | 470 | 51.8 | 437 | 48.2 |  |
|  |  |  |  |  |  |  |
| Cannabis use | Yes | 67 | 40.1 | 100 | 59.9 | **0.001** |
|  | No | 402 | 54.3 | 338 | 45.7 |  |
|  | Total | 469 | 51.7 | 438 | 48.3 |  |
|  |  |  |  |  |  |  |
| Other substance use | Yes | 20 | 51.3 | 19 | 48.7 | 0.950 |
|  | No | 448 | 51.8 | 417 | 48.2 |  |
|  | Total | 468 | 51.8 | 436 | 48.2 |  |

^a^ Based on *Chi^2^* statistic ^b^Row percentage
